# Personal Support Networks of Young People with Mild Intellectual Disabilities during the Transition to Adult Life

**DOI:** 10.3390/ijerph182211810

**Published:** 2021-11-11

**Authors:** Judit Fullana, Gemma Díaz-Garolera, Carolina Puyaltó, Ana Rey, Rosario Fernández-Peña

**Affiliations:** 1 Group of Research on Diversity, Institute of Educational Research, University of Girona, 17004 Girona, Spain; judit.fullana@udg.edu (J.F.); gemma.diaz@udg.edu (G.D.-G.); carolina.puyalto@udg.edu (C.P.); ana.rey@udg.edu (A.R.); 2 Faculty of Nursing, University of Cantabria, 39008 Santander, Spain; 3 IDIVAL Nursing Research Group, 39011 Santander, Spain; 4 SALBIS Research Group, University of León, 24400 León, Spain

**Keywords:** intellectual disabilities, personal network analysis, social networks analysis, social support, transition to adult life

## Abstract

Social support networks occupy a priority position requiring attention in the processes of social inclusion of people with intellectual disabilities, during their transition to adult life. The objective of the study was to analyze social support from a relational approach through Personal Network Analysis. A total of 41 young people with mild intellectual disabilities participated in the study, in two groups differentiated according to their educational stage, either compulsory secondary education or post-compulsory training. Descriptive and comparative results are presented based on the variables of structure, composition, and functional content in the social support of their personal networks. The results show that both groups have restricted personal networks, made up of members of the family and the educational environment who constitute the main providers of support. When moving towards adult life, the change in social contacts in other educational, geographical, and relational settings may mean a change in the provision of support received in previous life stages. Developing social and educational actions to support these people in the development and maintenance of social relationships is essential to their access to support resources that will affect their social inclusion.

## 1. Introduction

The social and relational environment in which people are immersed has received growing interest in the literature, due to its relationship with the health, well-being, and social inclusion of people with intellectual disabilities [1,2]. In the case of young people with intellectual disabilities, the literature has shown that these people tend to experience greater difficulties than their non-disabled peers in establishing relationships and that they tend to develop feelings of loneliness [3,4,5,6,7,8,9], their relationships are often limited to family members and professionals, and [10,11,12] they tend to find it difficult to maintain a stable group of friends during this vital stage [13]. In the study of social relationships, three aspects have been distinguished as follows: their existence and quantity, their formal structure, and their functional content, these are termed as social integration, social networks, and relational content, respectively [14].

Studies focusing on the social networks of people with intellectual disabilities of different ages have underlined the importance of improving social networks by promoting the development of new relationships through participation in the community, employment, school, leisure, and expanding ties with people outside the family group, since they can also be significant sources of social support [15,16,17,18,19,20]. A strong and stable social network throughout life enables people with intellectual disabilities to have the natural supports that may help them in their process of social inclusion [21,22]. Without well-developed social networks, it may be impossible for people with intellectual disabilities to develop in the processes of social inclusion [2,23].

The pathway toward an adulthood with autonomy and independence for young people with intellectual disabilities involves undergoing the transition to adult life. This is considered to be a multidimensional process, aimed at beginning working life, social and community participation, establishing satisfactory adult relationships, and beginning emancipation from the family [24]. Individual transition pathways that lead to the achievement of these objectives are built on the basis of support offered by schools and post-school services [25]. This process brings uncertainty, and new challenges appear in terms of social relationships [26], especially for young people with intellectual disabilities, who tend to experience a longer transition than young people without disabilities [27,28] and experience a progressive reduction in their circles of friendships [7]. During the transition to adult life, it is important to have a social support network made up of peers, since they can play an important role in the quantity and quality of support received [3,4,26].

Along these lines, the model for social inclusion of people with intellectual and developmental disabilities, by Simplican et al. [29], includes two interrelated domains that should overlap and mutually support one another: community participation and interpersonal relationships that are central to quality of life. 

Community participation is involvement in community activities that promote the development of interpersonal relationships. Successful community participation involves the building of linkages between community members in such a way that trust and positive social capital can be developed. This entails, among other factors, the participation in informal social mechanisms based on interaction with family, friendships, and neighbors, highlighting the importance of the characteristics of social networks in which the person is immersed [30,31]. Greater participation in the community should increase the person’s social network and, in turn, the strengthening of interpersonal relationships should increase their access and level of involvement in the community [29]. In contrast with wider community focused networks, restricted networks are typically small, with an absence of local kin, minimal contact with neighbors or wider community contacts, are heterogeneous and dispersed, and are characterized by more difficult access to support resources [32]. On the other hand, it is necessary to consider the function in interpersonal relationships to refer to the content that relationships can provide, such as social support. Interpersonal relationships present three types of characteristics to be considered, as follows: category, which refers to the kinds of people in the social network such as family members, staff, friends, acquaintances, and intimate partners; structure, which includes the length of the relationship, the frequency of contact, reciprocity, emotional closeness, size, homogeneity, and density, among other factors; function, which reflects the content that relationships can provide.

Social support is defined as “support accessible to an individual through social ties to other individuals, groups, and the larger community [33] and constitutes one type of functional content of social relationships [14]. In its theoretical and conceptual development, different dimensions of social support are present in the literature. This concept is typically divided into different types of support. Emotional support refers to relationships in which the person receives approval and acceptance from significant others, and they are esteemed and valued. Instrumental support—also termed tangible or material support—can include support in a wide range of activities or practical tasks. Informational support refers to a process through which other persons may provide information, advice, and guidance [34]. Other dimensions present in the literature on social support are the reciprocity or exchange that must be present for the support to continue [35] and the frequency of interactions that provide support [14,35,36].

Social support is a determining factor for the quality of life and the level of life satisfaction [37], in addition to being a key factor for the psychological well-being of children and young people with intellectual disabilities [38]. Different studies on the social support provided by the personal networks of people with intellectual disabilities [15,16,39] have shown that these people tend to receive less emotional support than people without disabilities. The studies show that this can cause feelings of loneliness and depression, and even lead to problems of mental health.

From a relational approach, in the study of social support is necessary to consider the characteristics of social networks since these provide the structural framework within which support may, or may not, be accessible to an individual [40].

Social support constitutes a resource derived from the relationships that make up a social network defined as a “set of actors and the ties among them” [41]. The study of these social networks constitutes one of the areas of application of the methodology based on social network analysis (SNA) [42], a research method that examines the interactions between individuals, groups, and organizations. One of the approaches of SNA is personal networks, which is the focus of this study. It forms a subset of the broader concept of egocentric networks and targets the relationships surrounding individuals in all the social environments to which they belong (e.g., family, co-workers, and neighbors) [43,44,45]. It has been used in studies on social support in chronically ill people and caregivers [46,47]. In the study of the personal network, the characteristics of the network structure, such as density, centrality measures, components, and isolates are distinguished (see [43] for further information regarding the variables), as well as the network composition, to refer to the characteristics of the network ties [48].

Thus, in the study of social support, SNA constitutes a suitable methodology for the study of the characteristics of the personal relationships proposed in the Simplican model [29], for the social inclusion of people with intellectual disabilities. With this approach, three differentiated aspects of social ties are distinguished, as follows: (a) the existence or number of relationships as a reflection of social integration; (b) the formal structure of social networks; (c) the functional content (in this case, social support) and the influence of the structure of social relationships on functional content in social support [49,50]. The dynamic nature of personal networks at all times reflects the result of a process of construction and re-composition that takes place over time [51]; therefore, life changes such as the end of studies, the entry into employment, or geographical mobility, act by modifying the form and structure of personal networks and, as a result, they show a construction elaborated over time [52]. In this way, the personal supportive relationships that these people have in the stage of transition to adult life occupy a priority position for attention due to their relationship with well-being and social inclusion.

Although different instruments have been developed to examine the characteristics of the social networks of people with intellectual disabilities, none of them examine the supportive relationships that exist between all members of the person’s network [53]. On the other hand, while there are some studies focused on the support networks of youth and adolescents with intellectual disabilities [6,54,55,56], most of studies on these networks have focused mainly on the stage of adult life [15,23,53,57]; therefore, there is a gap in the knowledge about support networks in people with intellectual disabilities in their transition to adult life. Therefore, the study presented in this paper focuses on the knowledge of social support from personal network approach during the transition period to adult life of people with intellectual disabilities. Due to the relevance of personal support networks in this vital stage, the objective of this study is to describe, analyze, and compare the characteristics of structure, composition, and function in the social support of the personal networks in two groups of young people with mild intellectual disabilities: one group is in the last stage of compulsory secondary education and another is in post-compulsory training programs (attention is aimed at the labor insertion of this group). 

## 2. Materials and Methods 

### 2.1. Design

This study was a descriptive, cross-sectional, and comparative study, using personal network analysis [45].

### 2.2. Sample Description

A total of 41 young people with mild intellectual disabilities (13 women and 28 men) participated in the study, selected through convenience sampling, and were divided into two groups. All the participants participating in the study had verbal communication and oral comprehension skills. The first group (school group, S) consisted of 27 participants engaged in compulsory secondary education, (18 in special education schools and 9 in mainstream secondary schools). Most of the participants in this group lived in the same town as their school or in towns close to their school. The second group (post-school group, PS) consisted of 14 participants who had completed compulsory schooling and were engaged in a training program for the transition to adult life. Most of the young people participating in the PS group resided in populations other than the city in which the organization provided the transition program. To access the participants in group S, the school directors acted as gatekeepers, before contacting their families. The participants of the PS group were accessed through the social worker of the organization that provided the training program. Schools helped in contacting with the students and their families to obtain their informed consent, and a social worker helped in contacting the participants in the transition to adulthood training program. These 41 participants reported 825 relationships that allowed analysis of the compositional and structural variables of their social networks, as well as their social support content.

### 2.3. Data Collection 

EgoNet open-source software (https://sourceforge.net/projects/egonet/ (accessed on 8 October 2018)) was used to collect each participant’s personal network data. Data collection from the S group was held between January 2019 and February 2020 and took place during school hours in a room provided by the school. Data collection from the PS group was held between March and May 2019 and took place in a room supplied by the service provider organization they attended.

Personal network data were collected through individual interviews based on an ad hoc questionnaire organized in four modules in accordance with the aim of the study, as follows:

First module: Information on the sociodemographic characteristics of each participant (ego).

**Figure 1:**
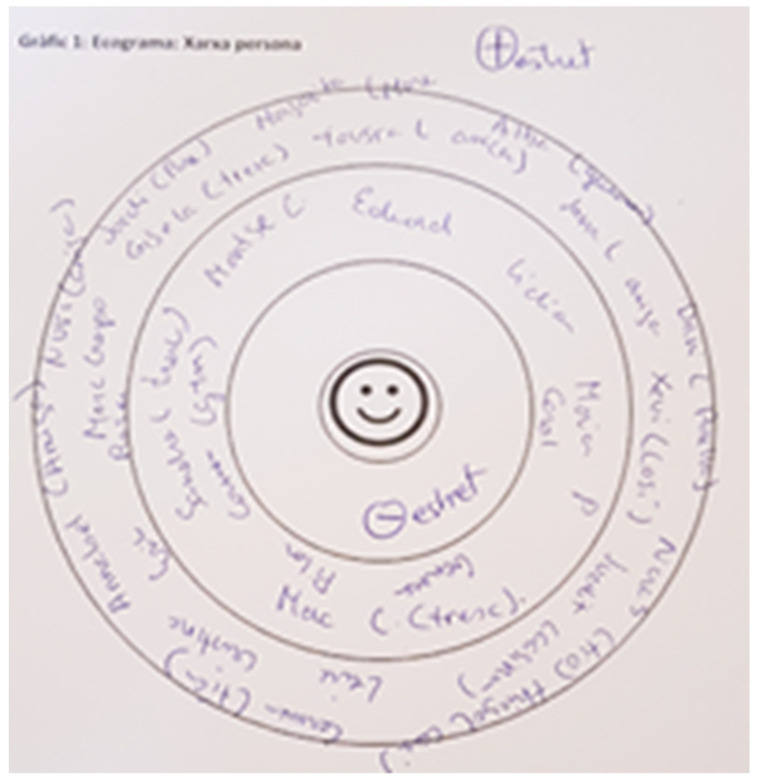
Ecogram (alters names).

Second module: Name generation question focused on identifying the people in their network (alters) belonging to the different areas of social life in which they are embedded (family, friends, fellow students, professionals, and neighbors, etc.). Participants were asked to name a fixed number of alters [58], specifically 25 alters, as this was considered an adequate number to obtain structural variables of the person’s network [59]. Nevertheless, many of them struggled to name 25 alters—most of them managed to name between 13 and 25 alters. Specifically, 14 participants (9 from the S group and 5 from the PS group) managed to name between 13 and 19 alters, and 27 participants named between 20 and 25 alters. To help them to identify their personal ties, an ecogram [16] was used as support material to help the participant identify their relationships (Figure 1).

Third module: Variables regarding the composition of the personal network (alter characteristics) and variables related to social support (social support function) were collected. 

**Figure 2:**
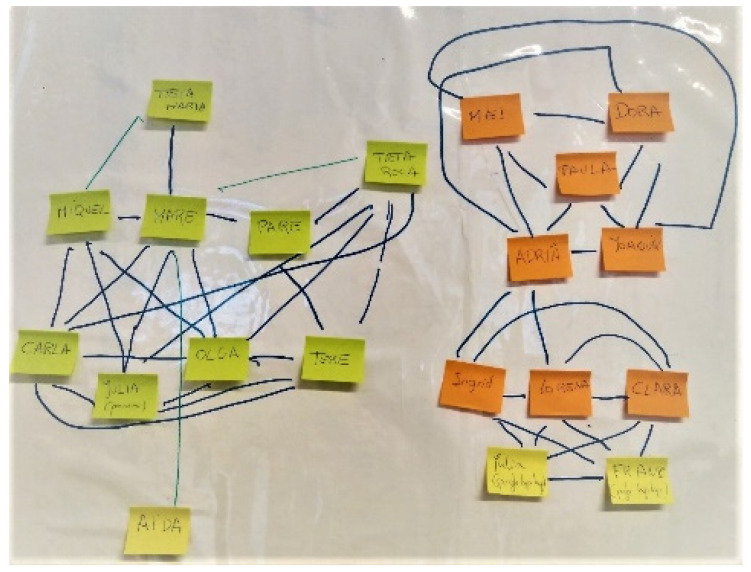
Handmade personal network (alters relationships).

Fourth module: Informants were asked about the relationship between possible pairs of actors among the contacts named. To help S group participants in answering this section, sticky notes were used to write down each of the names of their contacts (Figure 2), the names were placed on a board, and lines were drawn with markers to indicate the relationships between the contacts (Figure 2).

Data were collected by audiotaped interviews. They had a mean duration of 65 min (range 1–2 h). In the middle of the interview, the participants were offered the possibility of taking a break, which they accepted in most cases, especially the participants of the S group. Not all the participants answered all the questions. For example, many of them were not able to give the age of some members of their network, nor their occupation, among other information. All available data were analyzed, and in the results section, the number of real answers (N) is indicated for each variable. 

### 2.4. Variables

The study includes the following variables:

- Ego variables: age, sex, number of siblings, number of people cohabiting, and who they live with.
- Personal network composition (alter characteristics): age, sex, living place, tie with ego, main activity (study, work, a combination of both, or none of them), presence of disabilities, frequency of contact, variation of the relationship over the time, frequency of contact, place of relationship, and satisfaction with the relationship.
- Personal network structure: personal network data were obtained regarding density, degree centrality (mean), betweenness centrality (mean), components, and isolates.
- Social support variables (function): The participant was asked to answer questions about the social support characteristics of each of their alters: type of support received (emotional, instrumental, and informational), their possible combinations and no support to identify the non-support providers, support frequency, reciprocity in social support and setting in which the support took place. In the context of this study, emotional support referred to receiving help when they felt sad, angry or when they had to face a personal difficulty. Instrumental support referred to receiving help to do homework, housekeeping or other daily activities. Informational support referred to support received, to obtain and manage information related to school, homework, training program, travel infor-mation, etc. See Appendix A for more detailed information about the variables, categories, and the answers guide for the interviewer.

Personal network data were obtained through Egonet software. In addition, UCInet software [60] was used to obtain structural network measures. Statistical data sets for ego and alters characteristics were obtained. A descriptive analysis was conducted for informant’s characteristics, variables related to personal network structure, composition, and social support variables. Counts and percentages were calculated for category variables. Mean, median, minimum, maximum values, and standard deviation were estimated for quantitative variables. A comparison was made between the S and PS groups of the variables of composition, structure, and social support of personal networks. The chi-square test was used to compare category variables and the Student’s t test for independent groups was used to analyze quantitative variables (age and network structure variables). The values of these comparisons were considered significant for values of *p* < 0.05. All statistical analysis was performed using SPSS v.27 software (IBM SPSS Statistics, New York, NY, USA).

### 2.5. Ethical Considerations

The researchers applied for and were granted ethical approval, authorized by the Spanish State Research Agency who funded the project (protocol code EDU2017-84989-R, the date of approval was 14 June 2018). Accessible information about the research was provided to all participants and, once they agreed to participate, they signed consent forms. For participants under 18 years old, information about the research was also provided to their parents and guardians, who signed the consent forms. Data protection legislation was followed throughout the study (Spanish Organic Law on Data Protection 3/2018 and the Regulation (EU) 2016/679 of the European Parliament and the Council, 27 April 2016).

## 3. Results

### 3.1. Participant Characteristics

A total of 41 participants participated in the study (27 egos from group S and 14 egos from PS). A total of 825 relationships were obtained (545 alters in the egos of the S group and 280 alters in the egos of the PS group) that permitted the analysis of the compositional and structural variables of their personal networks. Table 1 synthesizes the characteristics of the mainly male participants in both groups. There was a mean age difference between the two groups of around 6 years, there were fewer partners in the PS group than in the S group, and some cases had life independence from the family nucleus in the older group. 

### 3.2. Descriptive Analysis

#### 3.2.1. Personal Networks Composition 

For the set of 825 alters, Table 2 presents the composition characteristics of the personal networks of the participants in both groups. 

Most of the relationships in the S group were made up of men, while in the PS group, women were more present. For the two groups as a whole, personal networks were made up of family members, friends, teachers, and other professionals (psychologists, support teachers, or professionals in extracurricular activities, etc.), as well as colleagues from their school or the center where they do the training. In both groups, what stood out was that around a third of their relationships were made up of people with intellectual disabilities, with their main relational environments being the educational centers and the home in which personal relationships take place on a daily or weekly basis. There was much less presence of their relationships in informal or leisure settings, especially for the PS group. In terms of duration, about a third of the relationships in both groups have been forged and have been present throughout their whole life. The comparison between the S and PS group highlights the fact that the egos of the latter group had a higher proportion of relationships initiated in the last two years (43.9% vs. 28.2%), who reside in more geographically distant places. 

Regarding the quality of the relationships—although in both groups they were generally satisfactorily valued, and the participants of the PS group valued an improvement in their relationships over time in a greater proportion than the participants of the S group (37.9% vs. 8.4%)—it is notable that in group S, the personal relationships that were valued as very satisfactory were double compared with those of the PS group. 

#### 3.2.2. Personal Networks Structure

The results for the personal network structure (Table 3) did not show significant differences between S and PS groups. However, in the comparison of means, the S group had greater intermediation or betweenness centrality than the PS group; on the other hand, in the networks of the egos of the PS group, there was a tendency to have more fragmented networks, with a greater number of isolates and components, than in the S group.

#### 3.2.3. Social Support Function

Of the 825 relationships studied, data were obtained from 811 relationships, of which 571 were identified as providing supportive relationships—71.9% of the relationships in the S group (389 relationships) and 67.4% of the relationships in the PS group (182 relationships). 240 relationships were identified as non-support providers, representing 28.1% of the relationships in the S Group (152 relationships), and 32.6% (88 relationships) in the PS Group (see Table 4). The non-support providers were men, for the most part, in both groups and had a lower mean age than the alters support providers in both groups (19.5 years vs. 24.9 years in the S group and 25.7 years vs. 32.2 years in the PS group). Regarding the geographic distance between the participants and their contacts, there were no statistically significant differences between providers and non-support providers in both groups; although, in S group, a higher percentage is observed—compared with non-support providers—of support providers who share a home with the ego. Regarding the link between ego and its contacts, non-support providers were found in all the links studied, including close relatives such as parents and siblings, in both groups. Support providers in both groups were concentrated in the family, followed by friends, classmates, teachers, and other professionals, with educational settings being the places where support was most present.

The most prevalent type of support in both groups, when it occurred in a unique way in relationships, was the emotional type, especially in the PS group (42.3% vs. 19.2%), followed by informational support (17.1% in both groups), and instrumental support, shown in a higher proportion in the S group than in the PS group (14% vs.7.4%) (see Table 5). However, the egos of the S group had providers of different types of social support in a higher proportion than the egos of the PS group (49.8% vs. 33.1%) and had supportive relationships with greater frequency and more reciprocal support than the egos of the PS group. Regarding the place where the support relationships take place, there were no significant differences between the two groups, with the home and the educational centers being where they mainly took place.

Regarding the distribution of the different types of support, according to the links with ego (see Table 6), the role of the family as a provider of support stands out, whether it presented in a single way or in combination with other types of support. However, in addition to family and friends, schoolmates, teachers, and other professionals occupied a priority place as providers of emotional support in the PS group.

#### 3.2.4. Personal Networks Visualizations

**Figure 3:**
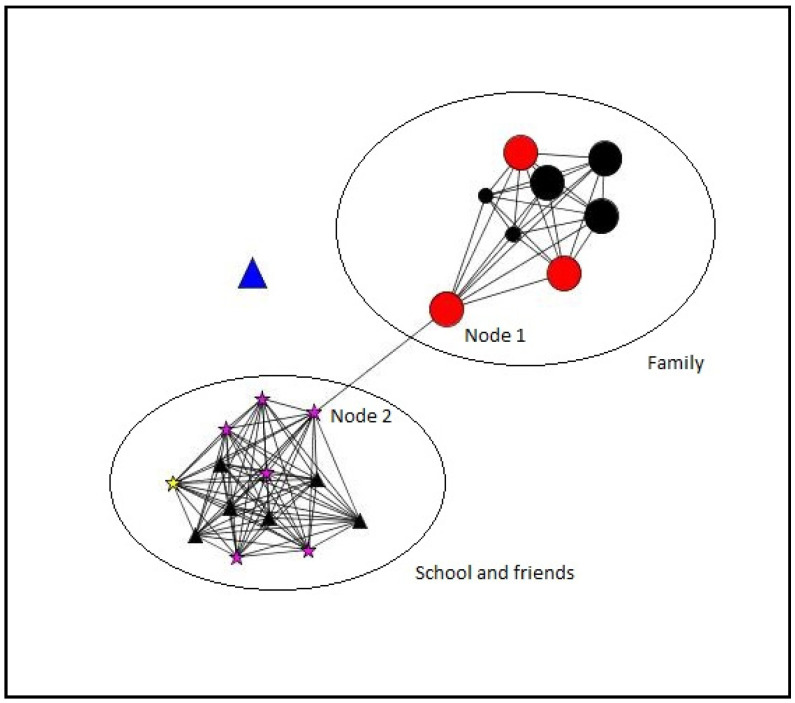
Case 1. Boy. 21 years old. Post-school group.

**Figure 4:**
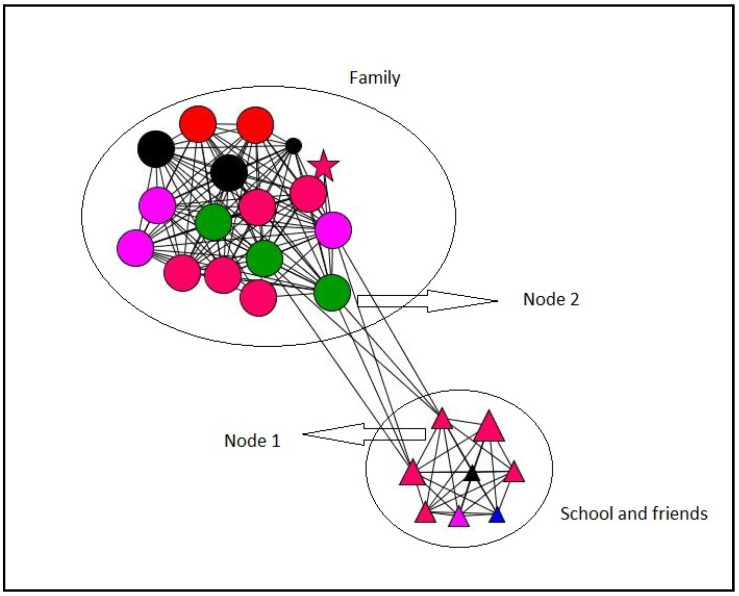
Case 2. Girl. 25 years old. Post-school group.

**Figure 5:**
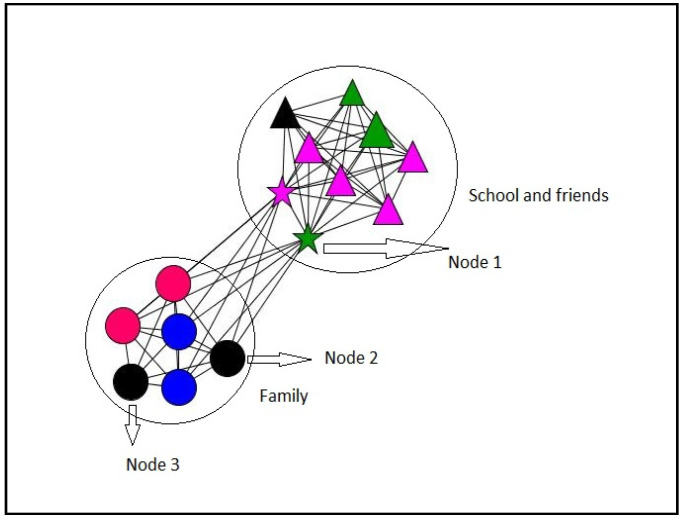
Case 3. Girl. 17 years old. School group.

**Figure 6:**
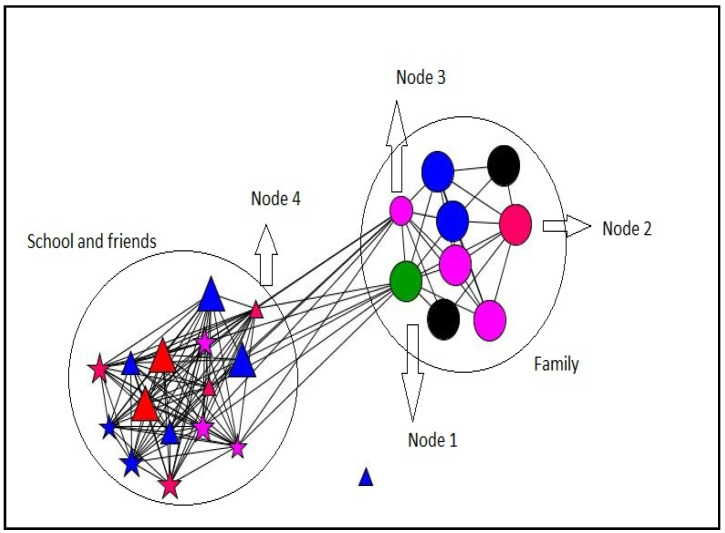
Case 4. Boy. 15 years old. School group.

In this section, some examples of visualization of personal networks are presented (Figure 3, Figure 4, Figure 5 and Figure 6). Graphs visually represent social networks. Each node in the graph identifies a person with whom the participant had a relationship, and the lines represent the relationships between them. The shape, color, and size of the nodes represent different characteristics of the alters (see Table 7). These case examples illustrate the different personal, relational, and supportive contexts in which these participants find themselves.

Case 1 (Figure 3) corresponds to a 21-year-old boy who belongs to the post-school group. He lives with his parents (currently unemployed) and his three siblings. In his support network, two large groups made up of family and classmates and professionals stood out—the latter with less time in relationships, due to the recent change in the educational environment and the formation of new relationships. Overall, a high proportion of non-support providers stood out in both social settings, the family roles being providers of emotional support, while, in the educational environment and that of friends, providers of more diverse or multiple support were predominant. The role of his father (node 1) and one of the institute’s educators (node 2) also stood out, showing the maximum intermediation between the group of alters, and, therefore, their ability to act as a bridge and connect between the two social environments was pronounced. 

Case 2 (Figure 4) corresponds to a 25-year-old girl who lives with her parents and has two brothers who live outside the family home. Again, in this case, the graph of her personal network shows two clearly differentiated groups belonging to the family and the educational environment, respectively. The latter has relationships created mostly in recent years; although, they are mainly emotional support providers. Due to its position in the structure of the personal network, node 1 stands out. This corresponds to a teacher with whom she began her relationship less than two years ago and who has the highest intermediation in the network, acting as a bridge between the educational and family environment. Likewise, the position of the mother (node 2), which has the highest degree centrality and closeness centrality in the network, stands out.

Case 3 (Figure 5) corresponds to a 17-year-old girl who has 5 brothers and lives with 4 of them and their parents. Regarding the functional content in social support of their personal network, nodes 2 and 3, corresponding to her father and brother and limited family support (both in number and type) stand out as non-support provider roles. However, the school environment and friends, with whom she has been in contact for several years, constitutes the main source of support, predominantly multiple and coming from her friends and classmates. Regarding the position in the network, node 1 has the highest centrality measures (betweenness, degree, and closeness) of all the members of the network and is, again, occupied by a teacher. 

Case 4 (Figure 6) corresponds to a 15-year-old boy who has a brother and whose parents (node 1 and 2) are separated. He lives with his brother and his mother’s partner (node 3). As in the previous cases, the personal network clearly shows two social settings, highlighting the mother’s position as a multiple support provider, indicating her position in the network with the highest value of betweenness. Node 4 is a friend who, despite having known her for a short time, has the greatest number of relationships and closeness to the rest of the network’s contacts (highest value of degree and closeness centrality). 

## 4. Discussion

This study has focused on analyzing the personal support networks in two groups of young people with intellectual disabilities who are in the process of transition to adult life, one group in the stage of completion of compulsory schooling and the other in a post-school training program. The results of the study have shown that the personal support networks of the participants in the two groups studied show differences, both in their composition and in their functional content in social support. 

In relation to their composition, in both groups, the personal networks were not very diverse in terms of types of ties, concentrating mainly on family, friends, schoolmates and some teachers and other professionals. The tendency to have restricted social networks was observed in other research carried out in adults with intellectual disabilities [15,61,62]. This characteristic may involve limitations in building the natural supports necessary to help the processes towards social participation [20] and social inclusion [21]. Among the differences in the composition of the personal network in both groups, it stood out that the members of the PS group had a higher proportion of relationships created in the last two years, while the S group had more relationships that have not changed over time. Likewise, the relationships of the PS group occurred less frequently than in the S group, and their contacts were more geographically distant. It was also observed in the study that the networks of the young people of the S group were a little more diverse in terms of composition than those of the PS group, in reference to the types of ties with alters. This is probably since, during the school stage, in addition to daily school attendance, they have more opportunities to participate in after-school and leisure activities in the community, which provide opportunities to develop social relationships. These characteristics may be a reflection of the changes in the contacts that take place in the transitions that occur in students when passing between different educational levels, as other studies have shown [63]. It is possible that the geographic and relational changes generated by the transition to a new educational environment in recent years in the PS group, as well as the beginning of an independent life from the family nucleus in some of the cases, at least partially justifies these differences.

The functional content in social support in both groups shows some differences as follows: of the total relationships studied, 71.9% were support providers and 28.1% were non-support providers, representing approximately one third of personal relationships. This is a similar proportion to that found in another study, performed in the context of chronic illness [46]. Non-support providers are mostly men, confirming the traditional role of women as providers of social support, as other studies have shown [47]. Regarding the type of support, the study shows that post-school participants perceived receiving more emotional support than participants engaged in secondary school, who perceived receiving more instrumental support. Research on the social support of adults with intellectual disabilities [15,19,39,64] show that they are less likely to receive emotional support than people without disabilities. This can lead to feelings of loneliness and depression. Emotional, instrumental, and informational support is provided, above all, by staff members of service providers and care professionals [23,61,62].This is the case because relationships with staff are one of the closest and most significant social relationships they have, so relationships with staff play a more central role in the lives of participants when they have few friendships or close relationships. In the study presented, the participants perceived that emotional support was provided by family and friends, which seems to indicate that, in the stage of transition to adult life—when the majority are still living in the family home and are engaged in school or in post-school training activities—professionals have a secondary role as providers of emotional support, and their role is perceived as providers of instrumental and informational support. This result differs from the studies mentioned that were carried out with adults, and they should lead to a reflection regarding the difficulties in maintaining natural relationships in adolescence that may be potential sources of support in adult life.

Although the family is the main source of support, educational environments are the settings where participants receive the most support; therefore, friends, classmates, and teachers or other professionals, especially the latter, become relevant providers in this context of multiple support, as other studies have shown [53,61,62,65]. These professionals from the institutions where people with intellectual disabilities are, represent the linking social capital, as they act as key intermediaries for obtaining resources that these institutions offer to individuals [18]. Their role is fundamental, especially in the group of seniors, in their transition to adulthood. Regarding the type of social support received, the geographical proximity [66] and the relationships maintained for a longer time throughout life were linked to the school in the S group. It is possible that this justifies the greater provision of multiple and more frequent support than participants in the PS group, who have already undergone a more significant change in their process of transition to adult life. 

Regarding satisfaction with personal relationships, although the participants of the PS group valued an improvement in their relationships over time to a greater extent, the egos of the S group valued their relationships as being more satisfactory than the egos of the PS groups. This is probably due to the frequency of their relationships, which is considered a determining factor for satisfaction with social networks, as the van Asselt-Goverts study has shown (among young adults with a mild to borderline [16] intellectual disability). In turn, frequent contact fosters shared values, increases mutual awareness of needs and resources, mitigates feelings of loneliness, encourages reciprocal exchanges, and facilitates the provision of support [67]. Likewise, these egos of the S group had more reciprocal relationships in support than the members of the PS group, which was an equally highly valued characteristic, as previous studies have shown [68,69].

Regarding the structural characteristics of the personal networks, our results did not show great differences between both groups. This was probably due to the limited number of participants, although this has served us well in exploring their characteristics. Taking the networks of the participants in the study together, the results obtained regarding the structural characteristics seemed to indicate that they were not very dense and not very cohesive networks. There were few people who had a role of intermediation between different groups of the same network, so it is possible that there is a redundancy of the resources available within the network and few possibilities of accessing new opportunities and support resources. Some studies suggest that geographic mobility, which in our study observed in the PS group, may have an effect on the lower cohesion of social networks [70]. Studies carried out with adults with intellectual disabilities show the difficulties in developing intermediation relationships that may favor social capital bridging [18].

The set findings seemed to indicate that, as one advances towards adult life, the change in social contacts when moving to other educational, geographical, and relational environments, may suppose, at least until the new relationships are consolidated, a change in the provision and characteristics of the support received in previous life stages. From an interventionist approach, the work of van Asselt-Goverts et al. [71] has shown that social network intervention in this group improves community participation, social networks, skills, and reduces loneliness. 

## 5. Conclusions

The analysis of personal networks facilitates the obtainment of indicators of structure, composition, and function in social support, as well as the changes that occur in different relationship contexts, due to transitions in different educational environments in the process of transition to adult life. Our results have shown changes in the composition of the personal network of the two groups, characteristics of personal relationships, and functional content in social support. Personal networks of young people with intellectual disabilities that are still attending school settings were a little more diverse in their composition than those of the young people attending a post-school program, but in both groups, the role of the family and educational setting as social support sources seemed clear. 

Moreover, the research findings point out that the opportunities to participate in different community environments does not necessarily increase when people grow up and leave compulsory school. The data about the length of the relationships indicate that these relationships are built in the context where people spend more of their daily time, and that the changes in life course mean having to start new relationships, which is not easy for people with intellectual disabilities.

To conclude, the findings of this exploratory research highlight the need to develop social and educational actions, from school and from post-school services, aimed at offering support to these people to develop and maintain social relationships that facilitate an increase in social capital and access to support resources. Both are essential to promote social inclusion. 

This research had some limitations. In the first place, in some cases, the participants with intellectual disabilities had difficulty in answering questions about their contacts, as well as in naming a fixed number of alters. Such information would have made it possible to compare and interpret the structural properties of personal networks. Secondly, the procedure followed in the sampling and access to the participants in an institutional educational context was mediated by the educational centers whose staff selected the cases and contacted the families and young people to present the research proposal. Finally, this study has focused on people with mild intellectual disabilities, so the voices of people with moderate or severe disabilities, or with difficulties in verbal communication (or both) have been excluded. 

Future longitudinal studies are necessary to account for the change experienced by people in the same cohort in this group during the transition to adulthood. Moreover, further research, based on personal network analysis, could deepen the comparison between people with and without intellectual disabilities, to the extent that this type of study may show possible differences in the structure and composition characteristics of personal networks in both groups, as well as their content in social support. These findings may illuminate the design of well-informed educational and social practices aimed at supporting the construction of social networks of people with intellectual disabilities.

## Figures and Tables

**Table 1:** Ego characteristics of the S and PS groups.

Variable		S *n =* 27	PS *n =* 14
Age	Mean	15.4	21.3
	SD	1.5	2.5
	Range	13–19	18–27
Sex	Men	19	9
	Women	8	5
Siblings	Mean	2	1.6
	SD	1.5	2.5
Number cohabiting	Mean	4.5	3.5
	SD	1.5	1.3
Who they live with	Parents	7	4
	Parents and siblings	17	8
	Parents and other relatives	3	0
	Flat mates	0	2

**Table 2:** Compositional characteristics of personal networks of the S and PS groups.

		S (545 Alters) *n* (%)	PS (280 Alters) *n* (%)	Total *n* (%)
Sex * n = 825	Women	141 (25.9)	106 (37.9)	247 (29.9)
	Men	404 (74.1)	174 (62.1)	578 (70.1)
Age * n = 636	N	479	157	636
	Mean	23.21	29.61	24.79
	SD	15.99	16.75	16.41
Living place * n = 752	Same home	75 (14.9)	37 (15.0)	112 (14.9)
	Same neighborhood	13 (2.6)	7 (2.8)	20 (2.7)
	Same town	179 (35.4)	35 (14.2)	214 (28.5)
	Same region	181 (35.8)	57 (23.1)	238 (31.6)
	Another region	41 (8.1)	107 (43.3)	148 (19.7)
	Another country	16 (3.2)	4 (1.6)	20 (2.6)
Activity * n = 785	Studying	306 (58.0)	97 (37.7)	403 (51.3)
	Working	181 (34.3)	121 (47.1)	302 (38.5)
	Studying and working	5 (0.9)	16 (6.2)	21 (2.7)
	Not studying not working	35 (6.6)	22 (8.6)	57 (7.2)
	Unemployed	1 (0.2)	1 (0.4)	2 (0.3)
Presence of Disabilities * n = 818	No	352 (64.9)	180 (65.2)	532 (65.0)
	Yes	148 (27.4)	91 (33.0)	239 (29.3)
	Don’t know	42 (7.7)	5 (1.8)	47 (5.7)
Tie *n =* 810	Parents	44 (8.2)	22 (8.1)	66 (8.1)
	Siblings	43 (8.0)	18 (6.6)	61 (7.5)
	Other relatives	82 (15.2)	61 (22.5)	143 (17.7)
	School mates	74 (13.7)	37 (13.7)	111 (13.7)
	Friends	195 (36.2)	79 (29.2)	274 (33.8)
	Partners in other activities	20 (3.7)	6 (2.2)	26 (3.2)
	Neighbors	2 (0.4)	0 (0.0)	2 (0.2)
	Teachers and other professionals	79 (14.6)	48 (17.7)	127 (15.8)
Variation of the relationship over time * n = 734	Has worsened	15 (3.3)	19 (7.0)	34 (4.6)
	Hasn’t changed	408 (88.3)	150 (55.1)	558 (76.0)
	Has improved	39 (8.4)	103 (37.9)	142 (19.4)
Length of the relationship * n = 806	Less than a year	54 (10.2)	40 (14.6)	94 (11.7)
	1–2 years	96 (18.0)	80 (29.3)	176 (21.8)
	2–4 years	123 (23.1)	36 (13.1)	159 (19.7)
	5–10 years	91 (17.1)	36 (13.1)	127 (15.8)
	Whole life	168 (31.6)	82 (29.9)	250 (31.0)
Frequency of contact * n = 809	Less than once a year	9 (1.7)	3 (1.1)	12 (1.5)
	1 or 2 times a year	26 (4.8)	13 (4.9)	39 (4.8)
	Every 2 or 3 months	23 (4.3)	18 (6.7)	41 (5.1)
	Monthly	43 (7.9)	22 (8.2)	65 (8.0)
	Weekly	119 (22.0)	147 (54.8)	266 (32.9)
	Daily	321 (59.3)	65 (24.3)	386 (47.7)
Place of relationship * n = 675	At home	98 (21.0)	59 (28.2)	157 (23.3)
	Educational center	229 (49.1)	92 (44.0)	321 (47.6)
	Extracurricular activities	16 (3.4)	9 (4.3)	25 (3.7)
	Leisure time activities	46 (9.9)	8 (3.8)	54 (8.0)
	Informal places	39 (8.4)	19 (9.2)	58 (8.6)
	Internet, phone	38 (8.2)	22 (10.5)	60 (8.8)
Satisfaction with the relationship * n = 818	Very unsatisfactory	7 (1.3)	11 (4.0)	18 (2.2)
	Quite unsatisfactory	51 (9.4)	26 (9.4)	77 (9.4)
	Satisfactory	283 (52.4)	187 (67.3)	470 (57.5)
	Very satisfactory	199 (36.9)	54 (19.3)	253 (30.9)

* Significance level *p* < 0.05.

**Table 3:** Personal network structure variables by groups.

	Group	*n*	Mean	SD	Min	Max	*t*	Sig.
Density	S	27	0.39	0.13	0.16	0.59		
	PS	14	0.40	0.14	0.22	0.74	−0.238	0.814
	Total	41	0.40	0.13	0.16	0.74		
Degree Centrality ^1^	S	27	7.08	2.13	3.06	10.80		
	PS	14	7.57	2.80	4.13	11.84	−0.567	0.577
	Total	41	7.25	2.36	3.06	11.84		
Betweenness Centrality ^1^	S	27	4.23	4.49	0.00	16.55		
	PS	14	3.61	3.62	0.00	9.44	0.477	0.636
	Total	41	4.02	4.18	0.00	16.55		
Components	S	27	1.59	0.50	1	2		
	PS	14	1.64	0.75	1	3	−0.227	0.823
	Total	41	1.61	0.59	1	3		
Isolates	S	27	0.52	1.16	0	5		
	PS	14	1.07	1.07	0	3	−1.525	0.138
	Total	41	0.71	1.15	0	5		

^1^ Mean: significance level *p* < 0.05.

**Table 4:** Providers and non-support providers by groups.

		S Non-Support Providers *n* (%)	S Support Providers *n* (%)	PS Non-Support Providers *n* (%)	PS Support Providers *n* (%)	Total Non-Support Providers *n* (%)
Sex *^,1,2^	Women	32 (21.1)	109 (28.0)	13 (14.8)	85 (46.7)	45 (18.7)
	Men	120 (78.9)	280 (72.0)	75 (85.2)	97 (53.4)	195 (81.3)
Age *^,1,2^	N	143	334	52	100	195
	Mean	19.5	24.9	25.7	32.2	21.1
	SD	14.9	16.2	15.9	17.0	15.4
Living place	Same home	14 (9.7)	61 (17.0)	8 (10.5)	29 (17.9)	22 (10.0)
	Same neighborhood	4 (2.8)	9 (2.5)	0 (0.0)	7 (4.3)	4 (1.8)
	Same town	57 (39.6)	120 (33.5)	15 (19.7)	18 (11.1)	72 (32.7)
	Same province	49 (34.0)	132 (36.9)	14 (18.4)	43 (26.5)	63 (28.6)
	Another province	13 (9.0)	27 (7.5)	35 (46.1)	65 (40.2)	48 (21.7)
	Another country	7 (4.9)	9 (2.6)	4 (5.3)	0 (0.0)	11 (5.0)
Tie *^,1,2^ *n =* 240	Parents	4 (2.7)	40 (10.3)	1 (1.3)	21 (11.5)	5 (2.2)
	Siblings	14 (9.6)	29 (7.5)	6 (7.5)	12 (6.6)	20 (8.9)
	Other relatives	29 (19.9)	53 (13.5)	21 (26.3)	36 (19.8)	50 (22.1)
	School mates	24 (16.4)	50 (12.8)	17 (21.3)	20 (11.0)	41 (18.1)
	Friends	59 (40.4)	133 (34.2)	30 (37.4)	44 (24.2)	89 (39.4)
	Partners in other activities	8 (5.5)	12 (3.1)	0	6 (3.3)	8 (3.6)
	Neighbors	1 (0.7)	1 (0.3)	0	0	1 (0.4)
	Teachers and other professionals	7 (4.8)	71 (18.3)	5 (6.2)	43 (23.6)	12 (5.3)

^1^ Significant differences between support providers and non-support providers of participants in group S. ^2^ Significant differences between support providers and non-support providers of participants in the PS group. * Significance level *p* < 0.05.

**Table 5:** Characteristics of social support received by groups.

		S *n* (%)	PS *n* (%)	Total *n* (%)
Type of support Received * n = 561	Emotional	74 (19.2)	74 (42.3)	148 (26.4)
	Instrumental	54 (14)	13 (7.4)	67 (11.9)
	Informational	66 (17.1)	30 (17.1)	96 (17.1)
	Emotional and instrumental	21 (5.4)	13 (7.4)	34 (6.1)
	Emotional and informational	62 (16.1)	7 (4.0)	69 (12.3)
	Instrumental and informational	57 (14.8)	21 (12.0)	78 (13.9)
	All types of support	52 (13.4)	17 (9.8)	69 (12.3)
Support Frequency * n = 400	Less than 4 times a year	31 (13.0)	42 (26.1)	73 (18.3)
	Monthly	44 (18.4)	26 (16.1)	70 (17.5)
	Weekly	109 (45.6)	72 (44.7)	181 (45.2)
	Daily	55 (23.0)	21 (13.1)	76 (19.0)
Reciprocity *n =* 547	No	87 (23.4)	44 (25.1)	131 (23.9)
	Yes	285 (76.6)	131 (74.9)	416 (76.1)
Place of relationship *n =* 484	At home	74 (21.6)	44 (31.2)	118 (24.4)
	Educational center	178 (51.9)	61 (43.3)	239 (49.4)
	Extracurricular activities	14 (4.1)	7 (5.0)	21 (4.3)
	Leisure time activities	33 (9.6)	7 (5.0)	40 (8.3)
	Informal places	21 (6.1)	11 (7.8)	32 (6.6)
	Internet, phone	23 (6.7)	11 (7.7)	34 (7.0)

* Significance level *p* < 0.05.

**Table 6:** Type of support received by tie for each group.

Tie	Group	Emotional	Instrumental	Informational	Emotional and Instrumental	Emotional and Informational	Instrumental and Informational	All 3 Types	Total (100%)
Family	S	25 (20.7)	23 (19.0)	14 (11.6)	7 (5.8)	22 (18.2)	9 (7.4)	21 (17.4)	121 (100)
	PS	29 (46.8)	6 (9.7)	6 (9.7)	7 (11.3)	1 (1.6)	4 (6.5)	9 (14.6)	62 (100)
Schoolmates	S	6 (12.0)	9 (18.0)	18 (36.0)	2 (4.0)	5 (10.0)	9 (18.0)	1 (2.0)	50 (100)
	PS	7 (35.0)	0	6 (30.0)	1 (5.0)	1 (5.0)	3 (15.0)	2 (10.0)	20 (100)
Friends	S	35 (26.3)	15 (11.3)	29 (21.8)	8 (6.0)	17 (12.8)	13 (9.8)	16 (12.0)	133 (100)
	PS	28 (63.6)	5 (11.4)	4 (9.1)	3 (6.8)	1 (2.3)	1 (2.3)	2 (4.5)	44 (100)
Partners in other activities	S	2 (16.7)	2 (16.7)	2 (16.7)	1 (8.4)	5 (41.7)	0	0	12 (100)
	PS	1 (16.7)	1 (16.7)	4 (66.6)	0	0	0	0	6 (100)
Neighbors	S	0	0	0	0	1 (100)	0	0	1 (100)
	PS	0	0	0	0	1 (100)	0	0	1 (100)
Teachers and other professionals	S	6 (8.7)	5 (7.2)	3 (4.3)	3 (4.3)	12 (17.4)	26 (37.7)	14 (20.4)	69 (100)
	PS	9 (13.4)	1 (5.4)	10 (11.6)	2 (4.5)	4 (14.3)	13 (34.7)	4 (16.1)	43 (100)

**Table 7:** Legend of graph.

Node Shape: Tie Type	Node Size: Relationship Time	Node Color: Social Support Type
Circle: Family Triangle: Schoolmates and friends Star: Teachers and professionals	Small node: less time Big node: more time	Emotional support: Red Instrumental support: Blue Two types of support: Pink All types of support: Green Non-support providers: Black

## Data Availability

Data belong the Group of Research on Diversity and owned by University of Girona and, therefore, under the University of Girona regulations as it contains potentially sensitive information on individuals. People interested in data of this study can contact to Judit Fullana, the main researcher of the study EDU2017-84989-R. Address: Pl. Sant Domenec, 9, 17004 Girona (Spain), e-mail: judit.fullana@udg.edu.

## Appendix A

**Table A1:** Social support function information: variables, questions, categories, and answers guide for the interviewer.

Variable/Question	Category	Answers Guide for the Interviewer
Type of support received Question: What type of social support or hep does this person provide you?	a. Emotional support	Answers about support related to love, empathy, trust, care, for example, referred to receiving help when the informant felt sad, angry or when he/she had to face a personal difficulty
	b. Instrumental support	Answers about receiving help to do homework, housekeeping or other daily activities like going to the doctor, or money administration.
	c. Informational support	Answers about support that helps to obtain and manage information related to school, homework, training program, travel information, or other valuable information to solve problems or making decisions.
	d. Emotional and instrumental e. Emotional and informational f. Instrumental and informational g. All types of support h. Doesn’t provide support	Select the options d, e, f or g in case the participant answers more than one type of support. Select option h in case the participant answers that the alter doesn’t provide social support.

Support frequency Question: How often does this person give you social support?	a. Less than 4 times a year b. Monthly c. Weekly d. Daily e. Doesn’t provide support	
Reciprocity Question: Do you provide social support to this person?	a. No	
	b. Yes	
Place of relationship Question: Where do you usually relate to this person?	a. At home	The place of relationship is the home where the participant lives
	b. Educational centre	The place of relationship is secondary school for S group and training program for transition to adulthood for Ps group.
	c. Extracurricular activities	The place of relationship is related to after school activities: sports, music, dance, arts, etc.
	d. Leisure time activities	The place of relationship is related to activities such as participation in hiker group, civic centre, playful activities, etc.
	e. Informal places	The place of relationships is street, park, bar, etc.
	f. Internet, phone	It is a non-face-to-face relationship
